# Competency categorization and roles of online teachers from the perspective of university students

**DOI:** 10.3389/fpsyg.2023.1009000

**Published:** 2023-03-02

**Authors:** Ragad M. Tawafak, Liqaa Habeb Al-Obaydi, Marcel Pikhart

**Affiliations:** ^1^Department of Information Technology, Al Buraimi University College, Al Buraimi, Oman; ^2^Department of English, College of Education for Human Sciences, University of Diyala, Baqubah, Iraq; ^3^Department of Applied Linguistics, Faculty of Informatics and Management, University of Hradec Králové, Hradec Králové, Czechia

**Keywords:** eLearning, hybrid learning, digital learning, Moodle, Google classroom

## Abstract

The teaching and learning process is facing many unprecedented challenges that require innovative solutions in the short life of knowledge and the abrupt development of technology. Some of these challenges are the new roles of teachers who are the main constituents in the online teaching process. The main aim of this study revolved around determining and analyzing university students’ priorities concerning the competency categorization and roles of online teachers. The research was based on Moodle and Google classroom to validate the competency elements with the final aim of improving teaching and learning processes. The data were collected by using an online questionnaire that evaluated eight dimensions of competencies and roles of online teachers. The research sample consisted of 430 participants (aged between 19 and 30) from Iraq and Oman. The results obtained from these two countries were very similar as the respondents highlighted professional, pedagogical and social competencies in their teachers. It further shows that though the applications used in the two contexts were different (Google classroom with Google Meet or Moodle), it did not affect the final results gained. The results of this survey could be important for further analysis of online teaching practice and bring several important insights regarding the possibilities of online teaching.

## Introduction

Traditionally, schools were based on the universal provision of education for all curricula and tests (Collins and Halverson, 2010). As experts, instructors were just reproducing verified knowledge that was required for the process of learning. The different subjects were taught as isolated disciplines and the technology employed in textbooks and whiteboards were determined (AlFarsi et al., 2021b). These schools that dealt with teaching young students and took the responsibility of future workplace employees have been generally accepted for school mission and they are unlikely to change even in the 21 century (Facer, 2011). Up to the middle of the 20th century, the growth of knowledge that works with changes showed a slow pace. However, this type of education gradually needed technological advancement, especially in information processing and communication between instructors and students, to cope with the requirements of the new digital era.

The limitations of traditional schools could inspire us to develop education through spotlights on emerging technologies for the future. In this context, this development reflects the change in educational practices moving toward technology-motivated and self-directed learning. That is why several new learning initiatives are gaining prominence in educational reform. The need to shift the traditional knowledge to modern developments was obvious, such as blended learning environments (Pikhart and Klímová, 2019; Al-Obaydi, 2021) or using Moodle or Google classroom in education (Tatnall, 2019; Tawafak et al., 2021a,b).

The fully online teaching process caused by the spread of the COVID-19 pandemic changed the teaching equation, putting different priorities for teachers and students in a new teaching situation (Tawafak et al., 2021b). On the part of students, the teaching process has to innovate new ways to develop students’ abilities to become knowledgeable, independent, creative, confident, and also become more democratic citizens (Kompri, 2015; Tawafak et al., 2021a). On the other hand, the transformation of teaching in the last 2 years from traditional, face-to-face teaching to an online or hybrid (or blended) education puts heavy emphasis on teachers to create new teaching strategies. It is also very important to note that in this context it is necessary to provide as many humanistic elements in their classes as possible (Al-Obaydi, 2021) because online teachers have to cope with this wave of development to manage and facilitate learning successfully.

To achieve this, Guasch et al. (2010) recommend using competencies in teaching online, which means “…a system of complex actions including the knowledge, abilities and attitudes that required for the successful completion of tasks” (p. 200). It is worth mentioning that the instruction is not enough for learners to be successful, especially when all the classes are conducted only online. There is also a need for many other roles and skills from teachers other than just teaching. The characteristics, functions, skills, and competencies the teachers need to gain are to be competent and qualified online instructors. All these aspects should be determined and highlighted by educational decision-makers, online learning theorists, and educational institutions. Faculty members require a clear framework and guidelines that help them improve their skills and support them in designing an accurate training approach (Munoz Carril et al., 2013). The dramatic change in education needs, as the first aim, to satisfy learners despite the differences in the contexts of learning and the electronic applications used. Therefore, it becomes essential to investigate teachers’ competencies in online education with the objective to see the learners’ choices regarding their priorities and to work extensively to satisfy students’ needs in this essential issue.

### The questions of the study

The present study intends to answer the following questions:

1. What are the leading roles that university teachers are supposed to play in online education from the students’ perspectives?
2. Are there any effects of an online application used in teaching on the arrangement of the roles?
3. Is there any effect of the geographical contexts of the study, i.e., Iraq vs. Oman, on the arrangement of the roles?

### The aim of the study

The main aim is to determine the university students’ priorities concerning the competency categorization and roles of online teachers in two different geographical contexts, i.e., Iraq and Oman.

## Literature review

Available literature proves that eLearning can offer an opportunity for universities to enhance teaching methodologies to improve learning outcomes in universities (Tawafak et al., 2018a,b; Pikhart and Klímová, 2020). Moreover, e-learning seems more suitable for higher education students who have experience using technology and are familiar with filling their needs (AlFarsi et al., 2021a,b). Researchers have argued that it is vital to know how students learn and collaborate in groups, programs, and courses to help students learning development (MacDonald et al., 2001; Engelbrecht, 2003; Ifinedo et al., 2018). Therefore, extensive efforts are needed to understand the adoption and implementation of continuous use of eLearning; however, certain gaps exist to enhance the continued use of eLearning and the knowledge on how teachers can satisfy these needs.

The recent developments in education call for the instructors to be experts with specific conditions to support the learning outcomes (Sugar and Warren, 2003). Still, there is a need to adopt various teaching technologies to support the instructors’ practices, whether using virtual or blended learning, specifically with the world’s current circumstances during the crisis of the COVID-19 pandemic and post-pandemic situation (Al-Obaydi, 2020). To systematize the instructor’s process various technological tools are used, such as the Moodle and Google classroom platforms. The educational models, as mentioned by Macdonald and Hursh (2006) are no longer adequate to be supported by workers or the ability to solve complex problems through confident exploitation of technology. It implies that students need to practice using technology tools and online resources to solve problems as individuals or groups within their experience (Howland et al., 2012; Tawafak et al., 2019). Nevertheless, this kind of virtual learning is still a challenge for university students and teachers in some areas of the world and using information and communication technology can be a challenge for them (Al-Obaydi, 2020).

The accelerated use of platforms in education impacted the assessment and teaching methods, teachers´ skills and practical usability. Students’ skills should be related to creativity, innovation, decision making, problem-solving and critical thinking to learn new knowledge and use it correctly (Tawafak et al., 2021b). Furthermore, more emphasis should be put on communication skills and collaboration between students. These new skills will impact the student’s mastery of writing, designing, and interacting with technology and their general improvements in the work context. Moreover, they also provide the ability to manage the information for practical work and valuable results as described when implementing the Moodle platform (Hafsa et al., 2021). On the other hand, the instructors need a lot of knowledge and experience to use electronic media smoothly and beneficially. These needs include technological knowledge, pedagogical knowledge, control knowledge, and technological content knowledge.

The advantages of using Google classroom and Moodle platform, as mentioned by Janzen (2014), are that they are simple and effortless to use. He adds that “Google Classroom’s and Moodle plan deliberately disentangles the directions interface and options used for conveying and following assignments; communication with the whole course or people is additionally streamlined through announcements, mail, and thrust notifications” (Janzen, 2014). They can also save time for the researcher, administrators, and advisor as they are planned to spare time. The apps can also coordinate and mechanize the use of other Google apps, including docs, slides, and spreadsheets. Chehayeb and Lienhard (2015) claims that Google is propelling a few highlights like trade grades to Google Sheets, more straightforward to overhaul review point scale, console navigation for entering grades, sort by title on evaluating page, etc., to spare teachers’ time. Cloud-based Google Classroom presents a more proficient and true innovation to utilize in the learning environment as Google apps speak to “a critical parcel of cloud-based undertaking communication apparatuses utilized all through the professional workforce” (Autret et al., 2016).

Another important aspect is flexibility. These apps are effectively available and useable to educate and learn in both face-to-face learning situations or in a fully online environment (Hafsa et al., 2021). It empowers teachers and evaluators to investigate and impacts “flipped directions strategies more effectively as well as automates and organizes the conveyance and collection of assignments and communications in different directions milieus” (Autret et al., 2016).

Another very important aspect is also that it is free. Google Classroom itself is not fundamentally accessible to learners without getting to an instructive institution. But anyone can get to all the other apps, such as Drive, Docs, Spreadsheets, Slides, etc., by signing up for a Google account. Mobile-friendliness is another crucial aspect as it is simple to utilize it on any portable gadget. “Portable get to learning materials that are alluring and simple to associate with is basic in today’s web associated learning environments.” (Janzen, 2014). Keeler et al. (2014) noticed a few other benefits of utilizing Google Classroom. She sees how Google Classroom ensures streamlined counseling by posting information, news and feeds for the students. That is why Stark and Crawford (2015) states that Google Classroom facilitates the use of collaborative learning. Here, the instructor can transfer materials and can provide criticism to the students who can transmit materials and make individual comments. Besides, the participants can collaborate by sharing their records and assignments. Keeler et al. (2014) also states that Google classroom encourages collaboration among students.

Working on the vision that teachers need to be successful in online education leads to many different viewpoints but one of the most useful could be that of Salmon (2003) who specified the qualities of online teachers in five points as follows: (1) personal characteristics, (2) technical skills, (3) understanding the online process online, (4) content expertise, and (5) communication skills. Alman et al. (2012) mentioned that distance teaching would allow teachers to learn about the features of instructional technology, emergent technologies needed, and online pedagogies. Researchers agree that teachers should have social, personal, pedagogical, and technical skills in addition to a set of abilities related to content, communication, management, and design (Guasch et al., 2010; Palloff and Pratt, 2011; Baran and Correia, 2014). More recent research by Albrahim (2020) concludes that teachers should have six categories of skills and competencies, including (a) content skills, (b) pedagogical skills, (c) design skills, (d) social and communication skills, (e) management and institutional skills, and (f) technological skills. Similarly, Abdous (2011) attempted to develop a framework by summarizing a set of competencies necessary before the teaching process including designing, preparing, and planning; during teaching, which includes providing, interacting, facilitating and seeking feedback; and, finally, after a lesson learned stage, which focuses on reflection on the lesson learned.

**Figure 1:**
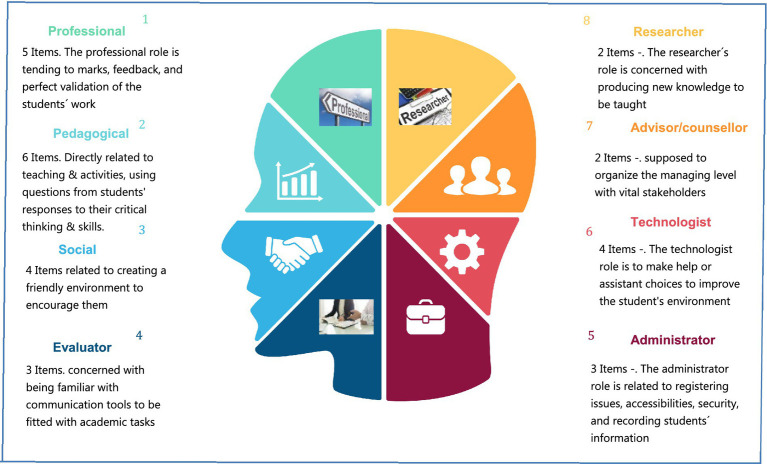
Research model of eight competency factors.

Bawane and Spector’s model (Bawane and Spector, 2009) which was used as a primary model in this study tried to elaborate competency categorization and the roles of teachers in online education creating one model. They constructed a comprehensive model based on the analysis of more than 10 researchers which consists of eight comprehensive roles: professional (role), pedagogical, evaluator, administrator, technologist, advisor/counsellor, and researcher. Each of these roles consists of sub-roles that cover all the details (see Figure 1).

Figure 1 shows the eight-competency categorization and functions based on Bawane and Spector’s model (Bawane and Spector, 2009) that will be used as a basic platform for this research study.

### Contexts of the study

The worldwide situation with the COVID-19 pandemic shows the impact that turned the whole world to online learning without any warning and a chance to prepare more systematically. Higher education institutions in Iraq and Oman, like in other countries, were forced to be completely online even there might have been a lack of suitable qualifications for fully online teaching and assessing process.

In Iraq, teaching in higher education institutions becomes fully online in the first year of the pandemic. Different types of online applications and platforms, including social media, were used in that year. The situation was challenging due to the lack of experience, therefore, it was a period of fear and anxiety for the scholars and decision-makers. After passing the first year, the ministry of education decided to apply online education for the second year. Distance learning was applied to all specializations in universities and colleges except human medicine. The platforms used were determined depending on the decision of each University council separately. At the University of Diyala, the students and instructors used Google Classroom and Google meet in teaching and examining. So, the current study deals with Google Classroom as a platform used in Iraq. The University of Diyala occurs in the Diyala governorate, situated in the middle of Iraq, characterized by its variety and diversity. It is a culturally diverse city with many cultures, sects, and nationalities (Al-Obaydi, 2019). The primary language is Arabic, and they use English as a foreign language.

In Oman, the same scenario happened with all institutions. They fully converted to online teaching as a second step after 1 month of entire lockdown which was a huge conversion from traditional classes and traditional paperwork of assignment submissions and exams. The new online teaching process started in April 2020. Most of the Omani universities used an official platform of Moodle and the use of social media as an alternative way to contact teachers and students. Distance learning applied in all universities except with medical colleges and some colleges that needed to use laboratory equipment. In Al-Buraimi University College, the students and instructors successfully accessed Moodle to go through all course’s requirements and Google meet for live sessions. Therefore, the current study focuses on Moodle as a platform used in Oman. The primary language used for communication and emails in English is Arabian, but there are many foreign instructors who speak English. Arabic languages are also used for law students and some general requirement courses such as Oman society, Islamic culture, and Arabic (Tawafak et al., 2018a).

## Method

### Instrument

To gain students’ perspectives on their teachers’ prominent roles in online education, the researchers constructed a questionnaire based mainly on Bawane and Spector’s model (Bawane and Spector, 2009). In this model, the roles of the teacher are specified by eight competency categorizations and functions, and they resulted in a comprehensive model. The model consists of these main dimensions: professional, pedagogical, social, evaluator, administrator, technologist, advisor/counselor, and researcher. To suit the contexts of the study, as this study applied in more than one context, the researchers modified some points of the model, for example, extending the items on each dimension to be more comprehensive—the data were collected by using an online survey method. In addition, the survey also contains the demographic information of the participants. The English language was used for the distributed questionnaire since it was directed to college students who can speak English. The data is analyzed using SPSS and Smart PLS 3.0 to answer the proposed research questions and to evaluate other necessary results of this study.

The questionnaire did not collect any personal data about the participants and their written agreements were obtained before the survey. The research was approved by the Ethics Committee of the University of Hradec Kralove no 2/2021.

### Participants

The number of students who took part in this study was 430 college students from Iraq and Oman, as shown in Table 1. An online survey was designed and distributed to them by Google Forms through emails. Different techniques and applications were used to distribute the online questionnaire officially and academically to the participants such as student university emails, Sending official link through Google classroom or through the use of Moodle platform to each participant individually. Table 1 shows the common demographics questions such as Age, Gender, Country and students’ perception to teach online, or physically with a teacher.

**Table 1:** Demographic information of participants.

Field	Demographic information	Number	Percentage
Total participant		430	100%
Age	19–22	280	65.1%
	23–26	66	15.3%
	27–30	38	8.8%
	More than 30	46	10.7%
Gender	Male	116	27%
	Female	314	73%
Country	Iraq	248	57.7%
	Oman	182	42.3%
Do you study in e-learning classes?	Yes	413	96%
	No	17	4%
Do you study with a teacher?	Yes	401	93.3%
	No	29	6.7%

### Validity and reliability

Bawane and Spector’s (2009) study highlights eight competencies and roles of the teachers in teaching online. However, their research did not present any results of their instrument’s statistical reliability and validity.

The survey feedback was tested through the SPSS program to identify the reliability impact. In this step, once the questionnaire was finalized, the next step was to test its reliability of the questionnaire using the Cronbach Alpha test. If the reliability passed Cronbach’s Alpha >0.7, the questionnaire would be distributed to the respondents.

The data collection was conducted online by the researchers in their sections at BUC and Diyala University. They were 182 undergraduate students from five areas in the Information Technology Department and 248 undergraduate students from four sections in the English department at Diyala University. The Google form survey link of the questionnaire was distributed during the participants´ class time.

Table 2 shows the validity of all received filled forms from 430 undergraduate students from Diyala University and Al Buraimi University College. 100% of them participated as the survey was built with compulsory answers to all items and sections without the possibility of leaving any. Table 3 shows Cranach’s Alpha = 0.979, which offers a high acceptance and reliability of the valid result.

**Table 2:** Case processing summary.

		*N*	%
Cases	Valid	430	99.8
	Excluded^a^	1	0.2
	Total	429	100.0

^a^Listwise deletion based on all variables in the procedure.

**Table 3:** Reliability statistics.

Cronbach’s alpha	Cronbach’s alpha based on standardized items	*N* of items
0.979	0.979	29

Accordingly, Table 4 presents the data analysis of questionnaire items, including question items, number, mean, standard error of the mean, median, standard deviation, variance, skewness and kurtosis. Concerning the mean, the value should be above 2.5, the median score should be 2.5, and the standard deviation value should be more than 0.5 to ensure that all item results can be accepted.

**Table 4:** Data analysis indicator of participants.

Question	Mean	Standard error of mean	Median	Standard deviation	Variance	Skewness	Kurtosis
Pro1	4.21	0.053	4.44	1.089	1.185	−1.225	0.609
Pro2	4.11	0.053	4.32	1.084	1.175	−1.098	0.458
Pro3	4.06	0.058	4.33	1.190	1.416	−1.105	0.192
Pro4	4.07	0.055	4.29	1.130	1.276	−1.104	0.432
Pro5	4.09	0.059	4.37	1.207	1.457	−1.140	0.233
Ped1	3.95	0.057	4.17	1.163	1.353	−0.906	−0.030
Ped2	4.08	0.053	4.28	1.086	1.180	−0.980	0.088
Ped3	3.95	0.054	4.13	1.117	1.247	−0.768	−0.304
Ped4	4.05	0.055	4.28	1.138	1.295	−1.140	−0.182
Ped5	4.07	0.057	4.33	1.170	1.368	−1.225	0.233
Ped6	4.05	0.058	4.31	1.195	1.429	−1.098	−0.162
Soc1	4.12	0.056	4.37	1.150	1.323	−1.140	0.192
Soc2	4.06	0.054	4.27	1.105	1.220	−1.098	−0.182
Soc3	4.13	0.055	4.37	1.132	1.282	−0.768	0.088
Soc4	4.10	0.054	4.32	1.114	1.240	−1.225	0.432
Eva1	3.94	0.058	4.16	1.182	1.398	−1.104	0.233
Eva2	3.92	0.057	4.17	1.195	1.398	−1.140	0.192
Eva3	4.00	0.053	4.28	1.150	1.353	−1.104	−0.182
Adm1	4.00	0.054	4.13	1.105	1.180	−1.225	−0.162
Adm2	3.96	0.055	4.28	1.132	1.247	−0.768	0.233
Adm3	3.96	0.057	4.33	1.114	1.295	−1.098	−0.030
Tec1	3.91	0.058	4.31	1.182	1.353	−1.140	−0.162
Tec2	4.02	0.056	4.37	1.195	1.180	−1.104	0.088
Tec3	4.06	0.054	4.27	1.150	1.247	−1.140	0.233
Tec4	3.99	0.055	4.37	1.105	1.295	−1.104	0.192
Adv1	3.94	0.054	4.32	1.132	1.276	−1.225	0.432
Adv2	4.05	0.058	4.16	1.114	1.457	−0.980	0.192
Res1	3.90	0.057	4.33	1.114	1.295	−1.098	−0.030
Res2	3.92	0.058	4.16	1.182	1.295	−1.104	0.192

## Results

According to the results achieved from the survey responses there was a high level of acceptance of all items with the values of mean, standard deviation, median, etc. (see Table 4). Therefore, the percentage of each category was to determine the highest influential factors that the student considered to be important when teaching online. The whole survey was analyzed with the SPSS program with significant acceptance results using a 5-point Likert scale (1 strongly agree to 5 strongly disagree).

However, the minor changes still could indicate the power and effectiveness of one role more than others. Table 5 shows the eight categories and functions used in the survey and the number of constructed items used to test its effectiveness. The summation of mean values from Table 4 is added then divided by the total number of items used in each role to find the highest category average.

**Table 5:** Data analysis of categories and roles.

Role	No. of items	Sum	Average
Professional	5	20.54	4.108
Pedagogical	6	24.158	4.026
Social	4	16.41	4.102
Evaluator	3	11.86	3.95
Administration	3	11.92	3.97
Technologist	4	15.98	3.99
Advisor	2	7.99	3.99
Researcher	2	7.82	3.91

Table 5 shows that three categories have the highest average of students’ acceptance and approval of the teacher’s competencies with online teaching, namely professional, pedagogical, and social, with high averages accounting for 4.108, 4.026, and 4.102, respectively.

The following items have a high mean value, as shown in Table 4. The professional role includes five things, were prof1 “Comply with ethical and legal standards,” Mean value = 4.21, and prof2 “Communicate effectively,” the mean value is 4.11 from both groups. The pedagogical role includes six items: five are above 4.00, and only one is less than 4.00. Social position is constructed by four things, three of them with high mean value, Soc1 “Maintain a cordial learning environment,” its mean value = 4.12. Soc3 “Refrain from undesirable behaviors,” the mean value = 4.13, and Soc4 “Promotes interactivity within the group” with a mean value = of 4.10.

The other five remaining categories also indicated good results. But the researcher wanted to find the item with the highest support.

Table 6 shows the correlations between items used in eight categories of the survey, and the diagonal gives a 1.00 correlation for each item with itself. All other correlations are above 0.5, which is correlated enough to be accepted (Tawafak et al., 2018a).

**Table 6:** Correlation between categories items.

	Pro1	Pro2	Pro3	Pro4	Pro5	Ped1	Ped2	Ped3	Ped4	Ped5	Ped6	Soc1	Soc2	Soc3	Soc4	Ev1	Ev2	Ev3	Adm1	Adm2	Adm3	Tec1	Tec2	Tec3	Tec4	Adv1	Adv2	Res1	Res2
Pro1	1.000	0.646	0.721	0.672	0.688	0.528	0.588	0.648	0.689	0.629	0.590	0.594	0.636	0.666	0.624	0.582	0.572	0.630	0.585	0.597	0.597	0.549	0.587	0.648	0.537	0.616	0.618	0.567	0.494
Pro2	0.646	1.000	0.701	0.679	0.696	0.650	0.580	0.668	0.732	0.665	0.642	0.609	0.660	0.575	0.629	0.642	0.564	0.558	0.613	0.575	0.625	0.576	0.617	0.638	0.589	0.686	0.554	0.599	0.517
Pro3	0.721	0.701	1.000	0.698	0.687	0.629	0.626	0.663	0.690	0.669	0.681	0.629	0.643	0.615	0.658	0.623	0.582	0.632	0.630	0.563	0.682	0.608	0.619	0.635	0.534	0.629	0.606	0.600	0.543
Pro4	0.672	0.679	0.698	1.000	0.702	0.633	0.624	0.664	0.723	0.686	0.660	0.665	0.639	0.653	0.673	0.634	0.623	0.591	0.605	0.549	0.634	0.546	0.629	0.581	0.568	0.592	0.627	0.557	0.527
Pro5	0.688	0.696	0.687	0.702	1.000	0.630	0.683	0.664	0.725	0.703	0.708	0.626	0.669	0.672	0.643	0.644	0.587	0.594	0.567	0.539	0.644	0.555	0.619	0.698	0.556	0.651	0.588	0.533	0.504
Ped1	0.528	0.650	0.629	0.633	0.630	1.000	0.632	0.733	0.629	0.667	0.595	0.578	0.606	0.548	0.602	0.639	0.562	0.579	0.571	0.516	0.550	0.564	0.574	0.580	0.569	0.649	0.606	0.539	0.522
Ped2	0.588	0.580	0.626	0.624	0.683	0.632	1.000	0.680	0.657	0.649	0.664	0.640	0.615	0.671	0.603	0.570	0.612	0.596	0.586	0.557	0.629	0.591	0.620	0.682	0.606	0.669	0.625	0.576	0.526
Ped3	0.648	0.668	0.663	0.664	0.664	0.733	0.680	1.000	0.674	0.660	0.663	0.594	0.662	0.623	0.655	0.626	0.613	0.627	0.639	0.557	0.652	0.622	0.633	0.634	0.559	0.710	0.639	0.598	0.535
Ped4	0.689	0.732	0.690	0.723	0.725	0.629	0.657	0.674	1.000	0.692	0.668	0.659	0.671	0.671	0.706	0.653	0.599	0.610	0.653	0.580	0.679	0.619	0.636	0.664	0.586	0.702	0.641	0.596	0.532
Ped5.	0.629	0.665	0.669	0.686	0.703	0.667	0.649	0.660	0.692	1.000	0.642	0.658	0.645	0.679	0.676	0.628	0.591	0.574	0.625	0.571	0.584	0.562	0.600	0.605	0.568	0.614	0.642	0.576	0.532
Ped6	0.590	0.642	0.681	0.660	0.708	0.595	0.664	0.663	0.668	0.642	1.000	0.664	0.665	0.662	0.652	0.650	0.592	0.592	0.667	0.558	0.663	0.616	0.638	0.650	0.516	0.655	0.626	0.495	0.579
Soc1	0.594	0.609	0.629	0.665	0.626	0.578	0.640	0.594	0.659	0.658	0.664	1.000	0.683	0.680	0.641	0.620	0.593	0.574	0.700	0.588	0.617	0.537	0.645	0.651	0.562	0.654	0.619	0.569	0.505
Soc2	0.636	0.660	0.643	0.639	0.669	0.606	0.615	0.662	0.671	0.645	0.665	0.683	1.000	0.695	0.666	0.624	0.651	0.584	0.642	0.612	0.654	0.577	0.684	0.636	0.613	0.671	0.654	0.561	0.549
Soc3	0.666	0.575	0.615	0.653	0.672	0.548	0.671	0.623	0.671	0.679	0.662	0.680	0.695	1.000	0.650	0.641	0.599	0.564	0.631	0.565	0.675	0.594	0.659	0.668	0.589	0.635	0.634	0.609	0.548
Soc4	0.624	0.629	0.658	0.673	0.643	0.602	0.603	0.655	0.706	0.676	0.652	0.641	0.666	0.650	1.000	0.640	0.578	0.625	0.600	0.561	0.659	0.559	0.605	0.660	0.601	0.653	0.605	0.576	0.583
Ev1	0.582	0.642	0.623	0.634	0.644	0.639	0.570	0.626	0.653	0.628	0.650	0.620	0.624	0.641	0.640	1.000	0.637	0.634	0.610	0.593	0.601	0.625	0.599	0.639	0.608	0.647	0.574	0.630	0.639
Ev2	0.572	0.564	0.582	0.623	0.587	0.562	0.612	0.613	0.599	0.591	0.592	0.593	0.651	0.599	0.578	0.637	1.000	0.618	0.618	0.558	0.584	0.581	0.620	0.554	0.551	0.613	0.578	0.599	0.538
Ev3	0.630	0.558	0.632	0.591	0.594	0.579	0.596	0.627	0.610	0.574	0.592	0.574	0.584	0.564	0.625	0.634	0.618	1.000	0.622	0.608	0.631	0.632	0.583	0.635	0.567	0.576	0.599	0.594	0.579
Adm1	0.585	0.613	0.630	0.605	0.567	0.571	0.586	0.639	0.653	0.625	0.667	0.700	0.642	0.631	0.600	0.610	0.618	0.622	1.000	0.635	0.682	0.608	0.637	0.649	0.613	0.695	0.653	0.588	0.560
Adm2	0.597	0.575	0.563	0.549	0.539	0.516	0.557	0.557	0.580	0.571	0.558	0.588	0.612	0.565	0.561	0.593	0.558	0.608	0.635	1.000	0.622	0.589	0.675	0.596	0.635	0.568	0.594	0.555	0.568
Adm3	0.597	0.625	0.682	0.634	0.644	0.550	0.629	0.652	0.679	0.584	0.663	0.617	0.654	0.675	0.659	0.601	0.584	0.631	0.682	0.622	1.000	0.674	0.634	0.715	0.577	0.671	0.649	0.624	0.602
Tec1	0.549	0.576	0.608	0.546	0.555	0.564	0.591	0.622	0.619	0.562	0.616	0.537	0.577	0.594	0.559	0.625	0.581	0.632	0.608	0.589	0.674	1.000	0.643	0.623	0.546	0.626	0.564	0.651	0.622
Tec2	0.587	0.617	0.619	0.629	0.619	0.574	0.620	0.633	0.636	0.600	0.638	0.645	0.684	0.659	0.605	0.599	0.620	0.583	0.637	0.675	0.634	0.643	1.00	0.622	0.653	0.656	0.633	0.591	0.590
Tec3	0.648	0.638	0.635	0.581	0.698	0.580	0.682	0.634	0.664	0.605	0.650	0.651	0.636	0.668	0.660	0.639	0.554	0.635	0.649	0.596	0.715	0.623	0.622	1.000	0.608	0.721	0.625	0.633	0.573
Tec4	0.537	0.589	0.534	0.568	0.556	0.569	0.606	0.559	0.586	0.568	0.516	0.562	0.613	0.589	0.601	0.608	0.551	0.567	0.613	0.635	0.577	0.546	0.653	0.608	1.000	0.633	0.579	0.630	0.663
Adv1	0.616	0.686	0.629	0.592	0.651	0.649	0.669	0.710	0.702	0.614	0.655	0.654	0.671	0.635	0.653	0.647	0.613	0.576	0.695	0.568	0.671	0.626	0.656	0.721	0.633	1.000	0.639	0.599	0.570
Adv2	0.618	0.554	0.606	0.627	0.588	0.606	0.625	0.639	0.641	0.642	0.626	0.619	0.654	0.634	0.605	0.574	0.578	0.599	0.653	0.594	0.649	0.564	0.633	0.625	0.579	0.639	1.000	0.547	0.596
Res1	0.567	0.599	0.600	0.557	0.533	0.539	0.576	0.598	0.596	0.576	0.495	0.569	0.561	0.609	0.576	0.630	0.599	0.594	0.588	0.555	0.624	0.651	0.591	0.633	0.630	0.599	0.547	1.000	0.683
Res2	0.494	0.517	0.543	0.527	0.504	0.522	0.526	0.535	0.532	0.532	0.579	0.505	0.549	0.548	0.583	0.639	0.538	0.579	0.560	0.568	0.602	0.622	0.590	0.573	0.663	0.570	0.596	0.683	1.000

Table 7 shows the significance of using two different variables, i.e., Iraqi and Omani students; each group used a different online platform model and different pedagogical methods. Also, Tables 8, 9 show a more diverse type of SPSS test to show the significant value of the items used in the survey.

**Table 7:** ANOVA with Tukey’s test for non-additivity.

			Sum of squares	df	Mean square	*F*	Sig
Between people			9980.976	419	23.821		
Within people	Between items		73.365	28	2.620	5.263	0.000
	Residual	Non-additivity	0.571^a^	1	0.571	1.147	0.284
		Balance	5839.789	11,731	0.498		
		Total	5840.359	11,732	0.498		
	Total		5913.724	11,760	0.503		
Total			15894.700	12,179	1.305		
Grand mean = 4.02							

^a^Tukey’s estimate of power to which observations must raise to achieve additivity = 0.608.

**Table 8:** Hotelling’s *T*-squared test.

Hotelling’s *T*-squared	*F*	df1	df2	Sig
104.004	3.475	28	392	0.000

**Table 9:** Intraclass correlation coefficient.

	Intraclass correlation^b^	95% Confidence interval		*F* test with true value 0			
		Lower bound	Upper bound	Value	df1	df2	Sig
Single measures	0.618^a^	0.585	0.651	47.851	419	11,732	0.000
Average measures	0.979^c^	0.976	0.982	47.851	419	11,732	0.000

A two-way mixed-effects model where people effects are random and measures effects fixed. ^a^The estimator is the same, whether the interaction effect is present or not. ^b^Type C intra class correlation coefficients using a consistency definition-the between-measure variance excluded from the denominator variance. ^c^This estimate is computed assuming the interaction effect is absent because it is not estimable otherwise.

## Discussion

The results of the present study show that there is a high level of acceptance among students of basically all items of the questionnaire as supported by the values of standard deviation, mean, median, etc. More specifically, the percentage of each category was determined to see the highest influential factors that gave student reflection on teacher competencies with online teaching. The discussions of the results in relation to each question of the study are as follows:

1. What are the leading roles that university teachers are supposed to play in online education from the students’ perspectives?

Results show that the primary roles of online teachers from the college student’s subjective point of view are professional, pedagogical, and social, respectively (see Table 6). This result indicates that it demands an experienced teacher to handle the new responsibility in teaching online professionally (Guasch et al., 2010), and to satisfy the needs of the students (Rad et al., 2022). During the time of COVID-19 when education turned to online teaching totally, the students in the whole world showed much more anxiety than before (Russell and Murphy-Judy, 2020). Klein et al. (2004) and Richey et al. (2005) attribute that the priority of professionalism is also crucial for online teachers. According to this study, professional roles, including the awareness of ethical and legal standards, can communicate effectively with students, demonstrate commitment, and can create a favorable positive class environment to break anxiety and support their opinions which is in agreement with (Alman et al., 2012). So, the role of the teacher is essential whether his/her rapport, credibility, or success in dealing with the students (Pishghadam et al., 2021).

The other competency, which was chosen by the respondents, is pedagogical. The pedagogical roles of online teachers are to design and prepare instructional strategies for the students, develop and use appropriate learning resources, use instructional methods in teaching, encourage participation among students, work on increasing students’ motivation and work on facilitating the study material (Pikhart et al., 2022). All these roles are essential according to the respondents’ answers. This result aligns with what is suggested by Dennis et al. (2004), who include communicational, technological, and discipline experts’ categories within the pedagogical one. Çakmak et al. (2021) also assessed the role of the teacher in EFL context and obtained similar results.

The third most prominent choice is the social one. To be a social teacher in online education is to ensure a friendly learning environment, prevent conflicts, refrain from unacceptable and bothering behavior, promote communication, and encourage speaking within the group (Derakhshan, 2021). The challenges that accompanied online education and the nature of presenting the material creates heavy emphasis on teachers to become social with their students and to help the students to interact and communicate in online classes, not just to become passive senders or receivers of knowledge. Though the social roles of online teachers seem critical in some contexts (Singleton, 2004), others agree on its importance to the students in motivating them towards learning, facilitating learning, and communicating effectively (Young, 2006).

The second most identified roles are technologist, advisor, administration, evaluator and researcher. While in Bawane and Spector (2009), the most identified roles were pedagogical, professional, evaluator, social and technologist roles.

2. Are there any effects of an online application used in teaching on the arrangement of the roles?

As far as the application used in teaching is concerned, it is crucial to say that though the application of the two countries differed, Google classroom with Google Meet in Iraq and Moodle in Oman, the results gained are similar to a large extent. It means that college students are aware of their needs and know well how to help themselves in such a situation.

3. Is there any effect of the geographical contexts of the study, i.e., Iraq vs. Oman, on the arrangement of the roles?

The similarity of the results in the two contexts proves that these roles (professional, pedagogical, and social) represent an absolute priority for college students (see Table 10) despite the geographical differences between the two samples (Derakhshesh et al., 2022). So, no clear affect was mentioned of the geographical context.

**Table 10:** Results analysis of three highest accepted roles.

Country	Role/Category	Sum of accepted answers	Average	Highest item	Numbers
Oman	Professional	713	142.6	Pro2	148
	Pedagogical	850	141.667	Ped5, Ped6	143
	Social	574	143.5	Soc1	146
Iraq	Professional	809	161.8	Pro1	171
	Pedagogical	895	149.167	Ped2	162
	Social	626	156.5	Soc1	163

**Figure 2:**
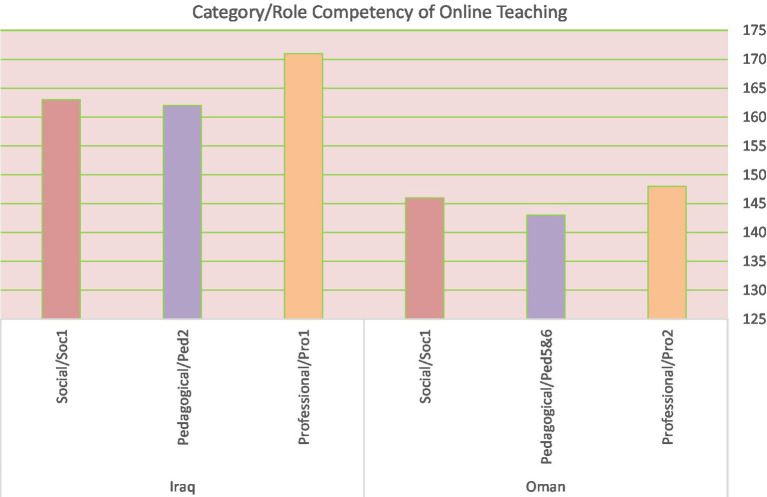
Category/role competency of online teaching.

It is worth mentioning that this research is the first that tests the effectiveness and validity of the category’s roles used in two different environments and different pedagogical methods of applying online teaching. However, Bawane and Spector’s (2009) study considered the average role ranks from expert feedback. On the other hand, this study used the norm of mean to determine the most influential category used in students who were forced to use online teaching without any previous training (Figure 2).

## Conclusion

The main conclusion of this study reveals that the professional role received the most priority amongst these eight identified sets of functions, followed by the social and pedagogical roles. These results are the same in the two countries even though the apps used were differing. The importance of the results gained from this study stems from the fact that students’ opinions prioritize determining the competencies, roles, and skills they need in their teachers to pass this difficult period of the pandemic. It can also be a road map for teachers in the present and upcoming times to develop their abilities. It’s as mentioned by Goold et al. (2010) that “even experienced teachers require support to continually adapt and improve their skills as the technologies continue to evolve” (p. 714). Thus, it is important to mention that training courses that cover technical knowledge and new methods of teaching for online teachers are essential. These courses can either be formal prepared by colleges and educational institutions and informal where teachers could attend them online (Debes, 2021). The issue of training leads to another point which is satisfying students’ needs and keep searching for more ways that can facilitate the process of leaning. The role of the teacher in a virtual classroom is crucial to students’ performance, and it calls for a slightly different skill set than learning in person, as recognized by today’s online educators. The teachers are aware that creativity, specialized training, subject understanding, and extensive preparation are the characteristics of a good online teacher. These results can help teachers, curriculum designers, and training committees to construct a clear vision of how to depend on competency-based teaching methods in online education.

Since the present research focused on only two Arabic Islamic countries may a limitation to the present study, it calls for more investigation to compare different contexts and use other models to obtain more global, reliable and systematic results about the roles of online teachers.

## Data availability statement

The original contributions presented in the study are included in the article/Supplementary material, further inquiries can be directed to the corresponding author/s.

## Ethics statement

The studies involving human participants were reviewed and approved by University of Hradec Kralove no. 2/2021. The patients/participants provided their written informed consent to participate in this study.

## Author contributions

All authors listed have made a substantial, direct, and intellectual contribution to the work, and approved it for publication.

## Funding

The publication of this article was funded by the SPEV 2023 Project run at the Faculty of Informatics and Management at the University of Hradec Kralove, Czech Republic.

## Conflict of interest

The authors declare that the research was conducted in the absence of any commercial or financial relationships that could be construed as a potential conflict of interest.

## Publisher’s note

All claims expressed in this article are solely those of the authors and do not necessarily represent those of their affiliated organizations, or those of the publisher, the editors and the reviewers. Any product that may be evaluated in this article, or claim that may be made by its manufacturer, is not guaranteed or endorsed by the publisher.
