# Supplementary material for: Competency categorization and roles of online teachers from the perspective of university students
Source: Front Psychol. 2023 Mar 2;14:1009000. doi: 10.3389/fpsyg.2023.1009000 (PMC10019092; doi:10.3389/fpsyg.2023.1009000)
Supplement: Supplementary file 1 [file Data_Sheet_1.docx]

***Appendix***

Competencies and roles of the online teacher questionnaire

This study deals with the main competencies and functions of teachers in an e-learning environment. So, if you join an e-learning class via Moodle or Google classroom, you are kindly requested to answer the following questionnaire:

Age:

1. 19-22
2. 22-25
3. 25-30
4. More than 30

Gender:

1. Male
2. Female

Do you study in e-learning classes? Yes/No

Do you check with a teacher? Yes/No

**Complete, please**

In an e-learning environment, the teacher should ……………….

| No | Items | Strongly agree | Agree | Partially agree | Disagree | Strongly disagree |
| --- | --- | --- | --- | --- | --- | --- |
| a | **Professional** |  |  |  |  |  |
| 1 | Comply with ethical and legal standards |  |  |  |  |  |
| 2 | Communicate effectively |  |  |  |  |  |
| 3 | Undertake efforts to update knowledge |  |  |  |  |  |
| 4 | Demonstrate commitment and favourable attitude |  |  |  |  |  |
| 5 | Break the anxiety of the students and create a positive class environment. |  |  |  |  |  |
|  | **Pedagogical** |  |  |  |  |  |
| 6 | Design instructional strategies. |  |  |  |  |  |
| 7 | Develop appropriate learning resources |  |  |  |  |  |
| 8 | Implement instructional strategies |  |  |  |  |  |
| 9 | Facilitate participation among students |  |  |  |  |  |
| 10 | Sustain students’ motivation |  |  |  |  |  |
| 11 | Facilitate the content material |  |  |  |  |  |
|  | **Social** |  |  |  |  |  |
| 12 | Maintain a cordial learning environment |  |  |  |  |  |
| 13 | Resolve conflict in an amicable manner |  |  |  |  |  |
| 14 | Refrain from undesirable behaviour |  |  |  |  |  |
| 15 | Promotes interactivity within the group |  |  |  |  |  |
|  | **Evaluator** |  |  |  |  |  |
| 16 | Monitor individual and group progress |  |  |  |  |  |
| 17 | Assess individual and group performance |  |  |  |  |  |
| 18 | Evaluate the course/program |  |  |  |  |  |
|  | **Administrator** |  |  |  |  |  |
| 19 | Manage the time and course |  |  |  |  |  |
| 20 | Demonstrate leadership qualities |  |  |  |  |  |
| 21 | Establish rules and regulations |  |  |  |  |  |
|  | **Technologist** |  |  |  |  |  |
| 22 | Access various technological resources |  |  |  |  |  |
| 23 | Select the appropriate resource for learning |  |  |  |  |  |
| 24 | Develop different learning resources |  |  |  |  |  |
| 25 | Suggest resources to the students (resource provider) |  |  |  |  |  |
|  | **Advisor/counsellor** |  |  |  |  |  |
| 26 | Suggest measures to enhance performance |  |  |  |  |  |
| 27 | Provide guidance based on student needs |  |  |  |  |  |
|  | **Researcher** |  |  |  |  |  |
| 28 | Conduct research on classroom teaching |  |  |  |  |  |
| 29 | Interpret and integrate research findings in teaching. |  |  |  |  |  |
